# Phosphatidylethanol in post-mortem brain: Correlation with blood alcohol concentration and alcohol use disorder

**DOI:** 10.1016/j.alcohol.2024.05.001

**Published:** 2024-05-17

**Authors:** Caine C. Smith, Julia Stevens, Mario Novelli, Dhiraj Maskey, Greg T. Sutherland

**Affiliations:** New South Wales Brain Tissue Research Centre, Charles Perkins Centre and School of Medical Sciences, Faculty of Medicine and Health, The University of Sydney, Camperdown, NSW 2006, Australia

**Keywords:** Alcohol use disorder, post-mortem brain tissue, phosphatidylethanol

## Abstract

Phosphatidylethanol (PEth) is an alcohol derivative that has been employed as a blood-based biomarker for regular alcohol use. This study investigates the utility of phosphatidylethanol (PEth) as a biomarker for assessing alcohol consumption in post-mortem brain tissue. Using samples from the New South Wales Brain Tissue Resource Centre, we analysed PEth(16:0/18:1) levels in the cerebellum and meninges of individuals with varying histories of alcohol use, including those diagnosed with alcohol use disorder (AUD) and controls. Our findings demonstrate a significant correlation between PEth levels and blood alcohol content (BAC) at the time of death, supporting the biomarker’s sensitivity to recent alcohol intake. Furthermore, this study explores the potential of PEth levels in differentiating AUD cases from controls, taking into consideration the complexities of diagnosing AUD post-mortem. The study also examined the relationship between PEth levels and liver pathology, identifying a link with the severity of liver damage. These results underscore the value of PEth as a reliable indicator of alcohol consumption and its potential contributions to post-mortem diagnostics and consequently, research into alcohol-related brain damage.

## Introduction

Human post-mortem brain tissue is an invaluable resource for neuropsychiatric research (Carlos et al., 2019). Pathological and molecular investigations of human brain tissues provide data crucial to understanding the complex aetiology of brain disorders. The findings from post-mortem brain studies not only augment clinical research and animal studies but can also inform the future directions of such research. The ongoing collection, storage, characterisation, and preparation of human brain tissue by purpose-built biobanking facilities is of vital importance to facilitate quality research.

The New South Wales Brain Tissue Resource Centre (BTRC), based at the University of Sydney, is a brain bank dedicated to collecting brain and spinal cord tissue from cases with alcohol use disorder (AUD), neuropsychiatric disorders, and healthy controls. Over 25 years, the BTRC has developed into an open-access biobank, distributing quality tissue and associated clinical information to hundreds of international researchers undertaking a wide variety of projects investigating the biology of alcohol use disorder. Every BTRC case undergoes extensive clinical and neuropathological review to determine appropriate case characterisation and assignment to a designated research cohort (Sutherland et al., 2016; Sutherland, Sheedy, & Kril, 2014). The accurate characterisation of each case is vital to appropriate cohort selection and data analysis validity. The process to determine the presence and severity of alcohol use in each case at the BTRC includes a review of available medical records and a questionnaire completed by a next of kin to inform a retrospective Diagnostic and Statistical Manual of Mental Disorders (DSM) diagnosis, neuropathological diagnoses, liver pathology and blood alcohol content (BAC) measures at the time of death. In cases where clinical information is limited or BAC is unavailable, the routine detection of a reliable biomarker of alcohol use would be a useful data point for characterisation purposes.

### Biomarkers of alcohol use

Measuring ethanol directly in exhaled breath, blood, or urine indicates recent alcohol intake and is detectable for up to 12 h before it is excreted or metabolised (Trius-Soler et al., 2023). Metabolic by-products, such as ethyl glucuronide and ethyl sulphate, are detectable for up to five days following alcohol intake in plasma and urine (Helander, Bottcher, Fehr, Dahmen, & Beck, 2009), and ethyl glucuronide remains detectable in hair for months (Crunelle et al., 2014). Fatty acid ethyl esters have been detected in blood plasma for up to 99 h (Borucki et al., 2007) and are also detectable in hair for months (Trius-Soler et al., 2023). There are non-specific markers of alcohol intake, such as carbohydrate-deficient transferrin, which is elevated following chronic alcohol intake, and gamma-glutamyl transferase, which is a liver enzyme that increases with liver damage (Nanau & Neuman, 2015).

Phosphatidylethanol (PEth) has emerged as a reliable indicator of medium-term alcohol use. In blood, PEth has a relatively long half-life of 4–10 days (Varga, Hansson, Johnson, & Alling, 2000; Weinmann, Schrock, & Wurst, 2016), can be detected between 3 days and 4 weeks after drinking (Gnann et al., 2010; Javors, Hill-Kapturczak, Roache, Karns-Wright, & Dougherty, 2016; Nguyen, Paull, Haber, Chitty, & Seth, 2018; Schrock, Thierauf, Wurst, Thon, & Weinmann, 2014; Schrock, Thierauf-Emberger, Schurch, & Weinmann, 2017; Wurst et al., 2015) and remains stable in preserved samples for years (Lakso, Wuolikainen, Sundkvist, Johansson, & Marklund, 2019). Under regular physiological conditions, phospholipase D catalyses the transphosphorylation of phosphatidylcholine to phosphatidic acid and choline (Gustavsson & Alling, 1987). In the presence of ethanol, PEth is produced with a phosphoethanol head group and two fatty acid chains corresponding to the parent phosphatidylcholine species. Currently, 48 homologues of PEth have been identified, with PEth(16:0/18:1) being the most abundant species in the blood (Gnann et al., 2010; Helander & Zheng, 2009; Nalesso et al., 2011). PEth(16:0/18:1) levels correlate well with self-reported total alcohol intake from the previous 2-weeks (Helander, Hermansson, & Beck, 2019).

Detection of PEth in tissues other than blood has been limited (Alling, Gustavsson, & Anggard, 1983; Aradottir, Lundqvist, & Alling, 2002; Aradottir, Seidl, Wurst, Jonsson, & Alling, 2004; Gustavsson & Alling, 1987; Lowery et al., 2018; Luginbuhl, Willem, Schurch, & Weinmann, 2018; Thompson et al., 2016). However, results from these studies indicate a positive relationship between alcohol use and PEth levels in serum and brain tissue at the time of death. This study investigates PEth levels in the post-mortem tissue of AUD cases and controls from the BTRC collection. The utility of PEth as a reliable marker of alcohol consumption around the time of death was explored.

## Materials and methods

### Cases

The use of human tissue for this study was approved by the University of Sydney Human Ethics Review Committee (HREC) (approval number HREC 2019/531). Informed consent from each brain donor was obtained through a prospective donor program or from next-of-kin via Forensic Medicine Sydney (HREC approval number 2019/531). Post-mortem unfixed, frozen brain samples were prepared according to our standard protocols (Sutherland et al., 2016). Each tissue sample was blinded using a unique identification number. Sample storage time was recorded, which refers to the time when the tissue was biobanked (i.e., stored at −80 °C) until the time it was retrieved for PEth(16:0/18:1) extraction. This ranged from 87 to 283 months. The case classification (meeting DSM-V criteria of alcohol use disorder (n = 46) criteria or not (n = 18) as well as the mean age, sex, brain weight and volume, body mass index (BMI), post-mortem interval, brain pH, and extent of liver pathology are reported in Table 1. Blood alcohol content was determined by NSW Forensic and Analytical Science Service as part of their autopsy procedure.

### Tissue preparation

Unfixed, frozen brain samples from the cerebellum and meninges were collected separately into 1.5 mL microcentrifuge tubes, weighed, and stored at −80 °C until required for lipid extractions.

### Chemicals and standards

All chemicals used for liquid chromatography-mass spectrometry (LCMS) were of high-performance liquid chromatography (HPLC) quality. *For lipid extraction*: ammonium acetate (Sigma-eAldrich, cat. no. 73594), methanol (Merck, cat. no. 1.06018), water (Merck, cat. no. 1.15333), methyl tert-butyl ether (SigmaeAldrich, cat. no. 34875), butylated hydroxytoluene (SigmaeAldrich, cat. no. B1378) and internal standard PEth(16:0/18:1)d5 (Echelon, cat. no. L-6051). *For liquid chromatography*: isopropanol (Merck, cat. no. 1.01040) and acetonitrile (AJAX FineChem^™^, cat. no. AJA2315) and external standard PEth(16:0/18:1) (Echelon, cat. no. L-6019).

### Lipid extraction

Twenty milligrams of tissue sample underwent a biphasic lipid extraction process using methyl *tert*-butyl ether, as described and validated in Matyash, Liebisch, Kurzchalia, Shevchenko, and Schwudke (2008). Briefly, tissue was homogenised using a pestle in the microcentrifuge tube the sample was collected in, along with 60 μL of 0.15 M ammonium acetate. 200 μL of methanol containing 10 ng of PEth(16:0/18:1)d5 and 0.015% (w/v) butylated hydroxytoluene and vortexed. 500 μL of methyl *tert*-butyl ether was added and sonicated in an ice bath for 30 min. 200 μL of water was added and incubated for 5 min at room temperature. Samples were then centrifuged at 1500×*g* for 5 min at room temperature. The lipid-containing upper phase was kept and dried in a vacuum centrifuge. Lipid pellets were re-suspended in 300 μL of methanol and aliquoted into autosampler vials (Waters, cat. no. 186002804) with screw-top lids (Waters, cat. no. 186000274) for chromatographic separation.

### LCMS

Lipids were separated by liquid chromatography using a Vanquish^™^ Duo UHPLC Systems (ThermoFisher Scientific) with an Accucore^™^ Vanquish^™^ C18+ UHPLC column 1.5 mm, 2.1 × 100 mm (ThermoFisher Scientific, cat. no. 27101–102130). The mobile phase A contained 20% isopropanol, 20% water, and 60% acetonitrile. The mobile phase B contained 40% isopropanol, 60% acetonitrile, and 5 mM ammonium acetate. A series of external standards containing 0.1 ng, 0.5 ng, 1 ng, 5 ng, 25 ng, 50 ng, 100 ng, 1000 ng, and 4000 ng of PEth(16:0/18:1) was prepared in 300 μL methanol, spiked with 10 ng of PEth(16:0/18:1)d5. Samples and standards were analysed by selected reaction monitoring in a TSQ Altis^™^ Plus triple quadrupole mass spectrometer (ThermoFisher Scientific). Double blanks containing only methanol were added between each sample. The negative precursor ions detected for PEth(16:0/18:1) was 701.512 *m/z*, and PEth(16:0/18:1)d5 was 706.6 *m/z*, both with two product ions: 281.292 *m/z* and 255.292 *m/z*.

### Data normalisation and statistical analysis

Data files were imported into Skyline 23.1 (Adams et al., 2020), open-source software supported by the National Institute of General Medical Sciences. Within the software, peaks for each sample were aligned and normalised to the PEth(16:0/18:1)d5 deuterated internal standard. These were plotted against the standard curve and exported to a spreadsheet where blank values were subtracted from each sample and normalised to tissue weight. R version 4.2.2 was used to conduct statistical analyses, including the Shapiro–Wilk test to assess the normality of each variable; the Wilcoxon rank-sum test with Bonferroni correction to investigate differences in variables between control and AUD groups; the Kruskal–Wallis test was used to assess whether there were overall differences in PEth(16:0/18:1) levels across the different levels of liver pathology, and subsequently a post hoc Dunn test for pairwise comparisons with a Bonferroni correction was applied to determine any significant differences in PEth(16:0/18:1) between the different liver pathologies. Graphs were created using GraphPad Prism 10.

## Results

### Relationship between PEth(16:0/18:1) levels and BAC

Cases across a range of drinking behaviours were compared between those who met or did not meet DSM-V AUD criteria. 63% of AUD cases had a positive BAC at death compared to only 28% of the non-AUD group (Table 1). In all cases with a positive BAC at the time of death, detectable levels of PEth(16:0/18:1) were observed in the cerebellum. Similarly, in the meninges, all but one case with a positive BAC showed detectable levels of PEth(16:0/18:1, with the exception having a very low BAC of 0.007 g/100 mL of blood. Low levels of PEth(16:0/18:1) were detected in 13 of the 17 cases with no BAC at the time of death. Only data for BAC-positive meninge samples were available for analysis (Table 2).

**Fig. 1.**
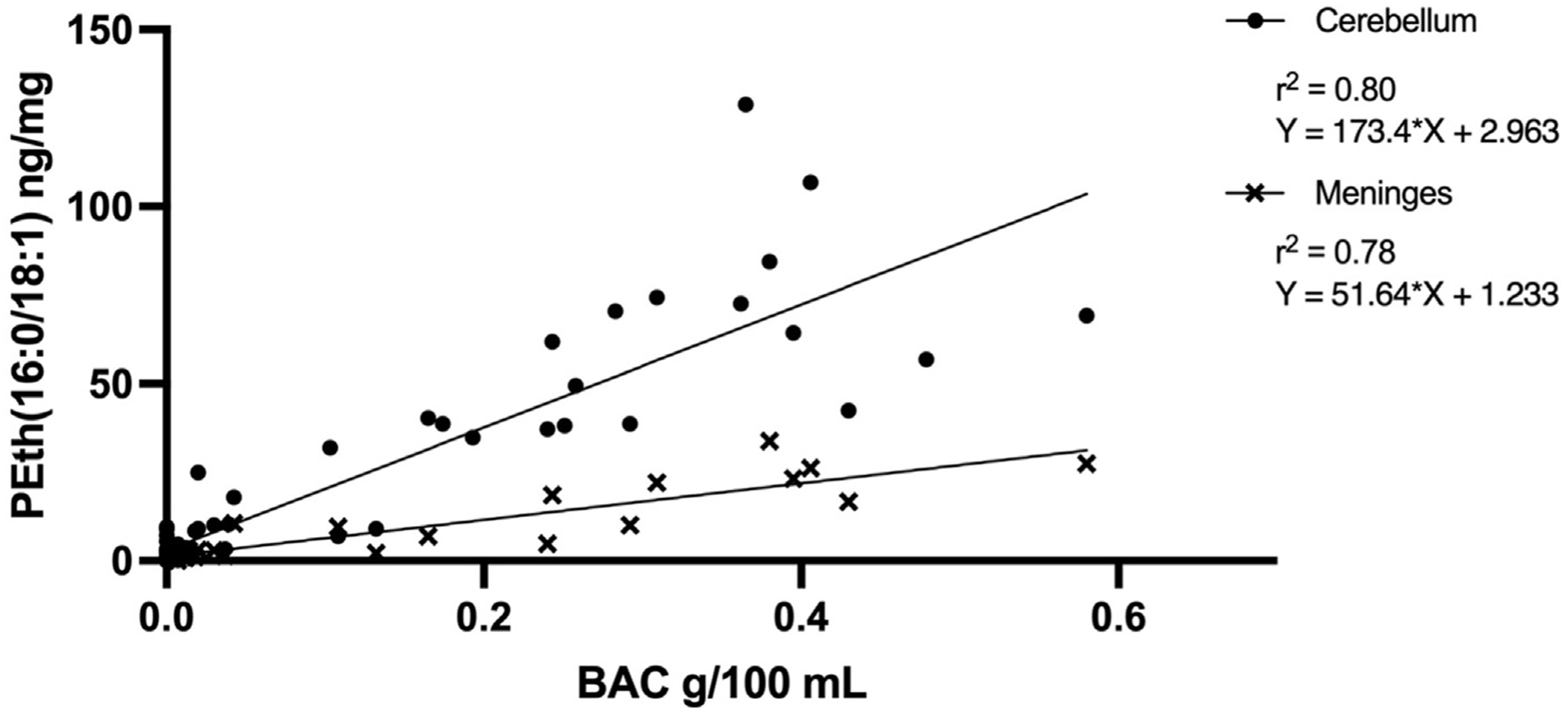
PEth and BAC. A scatterplot showing the relationship between PEth(16:0/18:1) levels in the cerebellum and the meninges with post-mortem BAC.

Regression analysis between PEth(16:0/18:1) levels in the cerebellum and BAC at the time of death showed a positive relationship (F(1, 62) = 242.349, p < 0.0001, r^2^ = 0.80). There was a similar relationship observed in PEth(16:0/18:1) levels in the meninges and BAC at the time of death (F(1, 20) = 67.90, p < 0.0001, r^2^ = 0.78) (Fig. 1). Due to less PEth(16:0/18:1) detected in the meninges than in the cerebellum for each case, and the lack of meningeal samples from BAC-negative cases, all analyses going forward are from cerebellar PEth(16:0/18:1) measurements only.

ANCOVA was used to test whether age, brain pH, PMI, sex, classification, sample storage time and BAC affected PEth(16:0/18:1) levels. Classification (i.e., AUD or control status) and BAC were the only significant covariates (F(1, 28) = 4.969, p = 0.03 and F(30, 28) = 155.7, p < 0.0001, respectively). Subsequent ANCOVA explored the interaction effects of BAC and the other covariates on PEth(16:0/18:1) levels. The only significant interaction was between BAC and brain pH (p = 0.04). Simple Slopes Analysis showed that at a higher brain pH and higher BACs, the predicted amount of PEth(16:0/18:1) decreases.

### PEth(16:0/18:1) as a biomarker for AUD

**Fig. 2.**
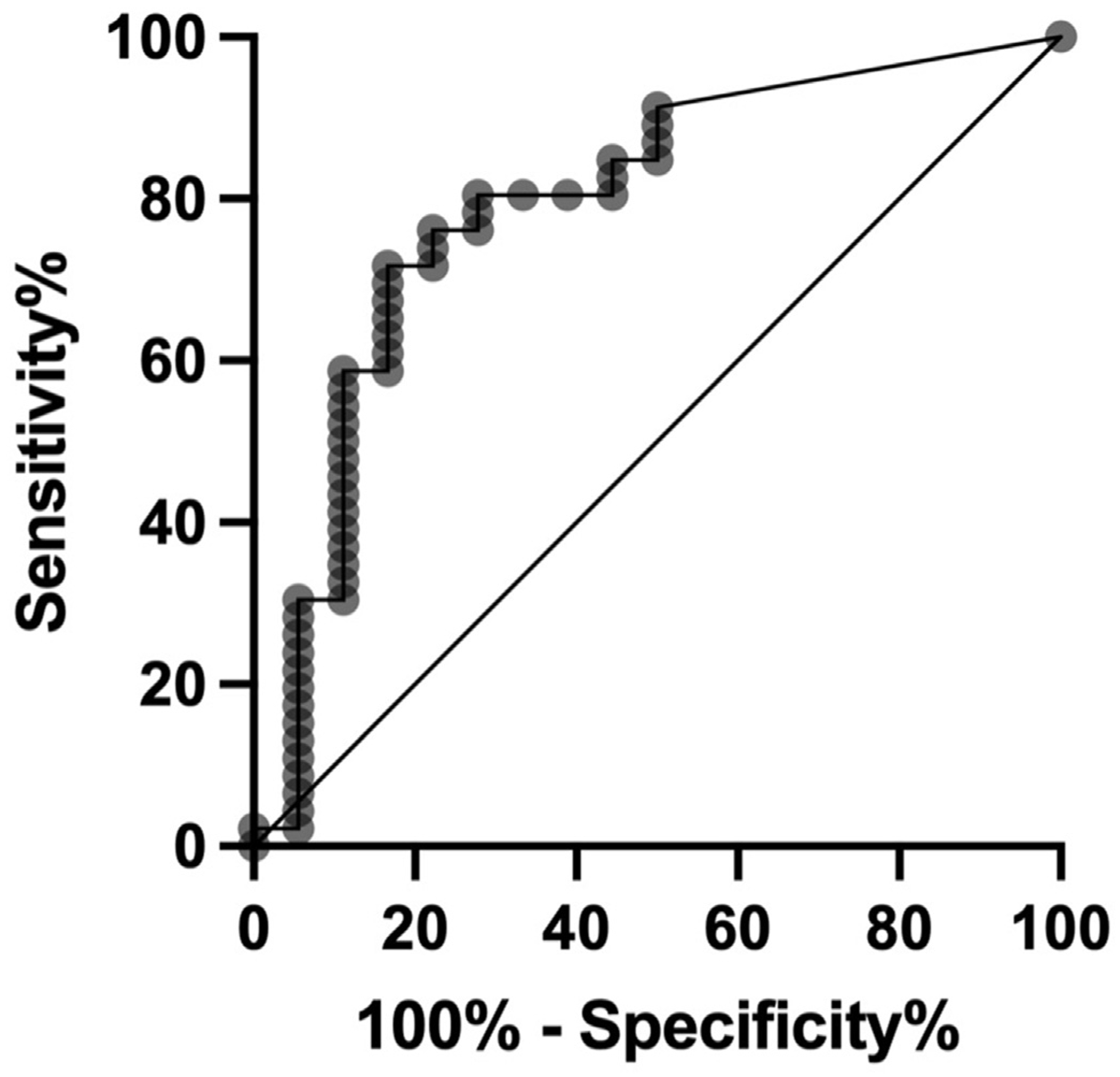
Predicting alcohol use disorder with PEth. A Receiver Operating Characteristic curve shows an area under the curve of 0.79 consistent with a high performance of PEth(16:0/18:1) concentration in predicting AUD status.

A Receiver Operating Characteristic (ROC) curve analysis was conducted (Robin et al., 2011) to evaluate PEth(16:0/18:1) as a biomarker for AUD, yielding an Area Under the Curve of 0.79 (Fig. 2). This analysis identified an optimal PEth(16:0/18:1) concentration cutoff of 3.05 ng/mg, demonstrating a sensitivity of 71.7% and a specificity of 83.3% for distinguishing between AUD and control groups. Subsequent logistic regression analysis, utilizing this cutoff to dichotomize PEth(16:0/18:1) concentration, revealed that individuals in this cohort with PEth(16:0/18:1) levels above 3.05 ng/mg were 12.7 times more likely to be classified as having AUD compared to those with levels below this threshold (OR: 12.8; p < 0.001; 95% CI: 3.1 to 51.3).

### Liver pathology

In assessing across, A KruskaleWallis rank sum test showed that PEth(16:0/18:1) levels were different depending on the degree of liver pathology in the donor (normal, steatosis, and cirrhosis; *X*^2^ = 8.2596, df = 2, p = 0.02). A Dunn’s test with a Bonferroni correction, showed that cases with liver cirrhosis had a significantly higher PEth(16:0/18:1) than brain tissue from donors with no liver pathology (Table 3).

## Discussion

The current study reinforces the role of PEth(16:0/18:1) as a robust biomarker for alcohol consumption (Helander & Hansson, 2023), due to its stability and detectability in the post-mortem context. A positive relationship between PEth(16:0/18:1) levels in the cerebellum and BAC at the time of death was established. PEth(16:0/18:1) levels above 3.1 ng/mg increased the likelihood of having a diagnosed AUD by 12.69 times. Cases that met the diagnostic criteria for AUD with liver cirrhosis tended to have higher levels of PEth(16:0/18:1).

The positive correlation of PEth(16:0/18:1) and BAC levels at post-mortem confirms the reliability of PEth(16:0/18:1) as a biomarker for recent alcohol use (Ulwelling & Smith, 2018). ANCOVA showed that neither post-mortem interval, nor storage time of the frozen brain tissue affected the levels of PEth(16:0/18:1), which confirms this as a stable biomarker (Lakso et al., 2019). BAC data is generally unavailable for brain donations to the BTRC unless toxicology investigations were performed by a Forensic pathologist at the request of the Coroner. Rather BTRC have relied on self-report and family recounts of the drinking behaviour of brain donors, which has varying levels of accuracy (Gilligan, Anderson, Ladd, Yong, & David, 2019). It is important to note that in this context, post-mortem BAC provides a biomarker of very recent alcohol consumption in the 24 h preceding death, whereas PEth(16:0/18:1), provides information on alcohol consumption in the weeks preceding death, which may be a more relevant marker for determining active drinkers.

The value of post mortem brain tissue for research is highly correlated to the amount of clinical information on a donor. Most of the AUD cases held by BTRC were consented retrospectively after entering the coronial system. Their clinical characterisation relies on next of kin surveys in combination with review of available medical records. This data is used for case classification and accurate cohort allocation, but may be incomplete relative to prospective donors (Sutherland et al., 2016). Under these circumstances PEth(16:0/18:1) provide an additional confirmation of recent alcohol consumption and the amount of alcohol consumed. An interesting aspect of the current study was an outlier in the control group, highlighting the complexity of interpreting PEth levels. This donor, with no prior history of alcohol misuse, succumbed to alcohol toxicity following a single heavy drinking session, which led to a very high BAC and PEth level at the time of death. This challenges the straightforward application of PEth as an AUD biomarker. It emphasizes the necessity of a nuanced approach in interpreting PEth levels, considering the individual’s clinical and consumption history, to accurately differentiate between acute intoxication and chronic misuse.

PEth has most commonly been detected in the blood, however it has also been detected in other tissues (Aradottir et al., 2004). Here we measured PEth in the cerebellum, as well as the meninges from the same cases that were BAC-positive only. Our findings revealed less PEth detected in the meninges, however, the pattern of the levels of PEth was similar i.e., higher levels of PEth in the cerebellum were mirrored by higher levels of PEth in the meninges. This variation in PEth concentration between the meninges and the cerebellum could be attributed to differences in cellular composition, ethanol exposure, blood supply, and enzymatic activity, notably phospholipase D, which is not uniformly expressed (Barber et al., 2022). Future studies should establish a better link between PEth levels in the cerebellum and the blood, to improve our understanding of the distribution of PEth and its metabolism in the body.

To further our insights into alcohol misuse and post-mortem case classification, future investigations should consider other variables that may influence PEth levels, including genetic predispositions, metabolic differences, impact of medications (Krzystanek, Krzystanek, Skalacka, & Palasz, 2020), and concurrent substance use. Expanding the scope of post-mortem laboratory tests to encompass these variables will significantly improve our ability to model alcohol misuse, offering a more nuanced approach to understanding its effects in the absence of a clinical diagnosis. This comprehensive approach will be instrumental in refining our strategies for assessing and classifying alcohol-related pathologies post-mortem, paving the way for more accurate and informative research in the area.

## Figures and Tables

**Table 1:** Brain donor characteristics summary.

Characteristic	Positive BAC	Negative BAC
Total number of cases (n)	34	30
Alcohol use disorder	*29*	*17*
No psychiatric diagnosis	*5*	*13*
Age (mean ± SEM; range)	50.1 ± 1.8; 25–71	57.1 ± 2.0; 33–73
Male/female	26/8	21/9
Post-mortem interval (hours) (mean ± SEM; range)	36.4 ± 2.3; 3–61	31.4 ± 2.7; 2–59
Brain pH (mean ± SEM; range)	6.59 ± 0.05; 5.92–6.95	6.55 ± 0.05; 5.82–6.95
Blood alcohol content (g/100 mL; mean ± SEM; range)		Not detected
Alcohol use disorder	0.19 ± 0.03; 0.007–0.58	
No psychiatric diagnosis	0.16 ± 0.08; 0.007–0.41	

**Table 2:** Cases with detectable PEth(16:0/18:1).

	BAC positive	BAC negative
Cerebellum		
PEth(16:0/18:1) detected	34	17
PEth(16:0/18:1) not detected	0	13
Meninges		
PEth(16:0/18:1) detected	21	n/a
PEth(16:0/18:1) not detected	1	n/a

**Table 3:** Significance of liver pathology.

	Z	Adjusted p-value
Normal vs. Steatosis	−1.825	0.10
Steatosis vs. Cirrhosis	1.416	0.24
Normal vs. Cirrhosis	2.845	0.007
